# Strain echocardiography in predicting LV dysfunction in RV apical pacing

**DOI:** 10.1016/j.ihj.2023.01.001

**Published:** 2023-01-03

**Authors:** Goutam Datta, Dipankar Ghosh Dastidar, Hrishikesh Chakraborty

**Affiliations:** Department of Cardiology, Burdwan Medical College, Burdwan, India

**Keywords:** Strain Echocardiography, RV apical pacing, LV dysfunction, Heart failure, GLS, GLS - Global Longitudinal strain, STE - Speckle Tracking Echocardiography, PICMP - Pacing induced Cardiomyopathy, PIVD - Pacing induced ventricular dysfunction

## Abstract

Right ventricular (RV) pacing is associated with a reduction in left ventricular (LV) systolic function, thought to be mediated by pacing-induced ventricular dyssynchrony. The prevalence of heart failure after RV pacing is reported to range from 31±3%. We studied 60 subjects with high-grade atrioventricular block and Complete Heart Block (CHB) scheduled to undergo right ventricular apical pacing. 2D echocardiography was done at baseline, 1 month and 12 months. Pacing-induced cardiomyopathy was defined as a reduction in LVEF to <45%. Strain was evaluated off-line from digitally stored images using all advanced software package (cardiac wall motion quantification (CMQ); Toshiba Medical Systems). Longitudinal strain for individual myocardial segments was measured from the apical four-chamber, two-chamber and long axis views (16 segment AHA/ASE model). None had LV dysfunction at baseline based on 2D and strain echo imaging. Subsequently 18 patients were detected to develop low GLS score (less than -14.5) at 1 month. On subsequent follow up at 1 year, all 18 patients developed LV dysfunction on 2D Echocardiography. Thus Strain imaging with GLS score helped in early detection of LV dysfunction in RV apical pacing subjects. Pacing-induced cardiomyopathy had significant association with high grade AV block with pacemaker dependency. It had no significant associations with other comorbidities like diabetes, hypertension, ischemic heart disease or with the type of medication intake. However there was a statistically significant association with heart failure.

## 1 Introduction

Right ventricular (RV) pacing is associated with a reduction in left ventricular (LV) systolic function,^1^ thought to be mediated by pacing-induced ventricular dyssynchrony.^2^^,^^3^ The prevalence of heart failure after RV pacing is reported to range from 31 ± 3%, the variation largely depending on the definition and methods used to define heart failure as well as the population examined.^4^, ^5^, ^6^, ^7^, ^8^, ^9^

Randomized trials in RV paced individuals have shown a near 3-fold increased risk of hospitalization for heart failure when the cumulative percentage of ventricular pacing (Cum%VP) exceeds 40%.^5^^,^^10^

The term pacing-induced cardiomyopathy (PICMP) has been used to describe clinically significant left ventricular systolic dysfunction (ejection fraction < 45%) attributable to RV pacing, occurring in the absence of other causes of cardiomyopathy.^4^^,^^7^ However, lesser degrees of pacing-induced LV dysfunction (PIVD) have also been observed in up to two-thirds of patients with normal baseline LV function.^3^^,^^11^^,^^12^ Despite this, clinical guidelines do not currently recommend routine cardiac imaging after pacemaker implantation, and PICMP/PIVD may therefore go undetected until the onset of heart failure symptoms.^8^

In this background, an early identification of patients at risk of developing heart failure after RV pacing would be of considerable clinical value as these patients may benefit from heightened clinical surveillance and possible upgrade to biventricular pacing, which has been shown to reverse PIVD and LV remodeling.^13^, ^14^, ^15^ A non-invasive test able to identify such individuals is, therefore, highly desirable. Two-dimensional (2D) speckle tracking strain echocardiography (STE) has been shown to detect early signs of LV systolic dysfunction before a measurable reduction in LVEF. The objective of the present study was whether STE can be used to predict the development of PIVD and PICMP after RV apical pacemaker implantation.

## 2 Materials and methods

The present study was an investigator-initiated prospective observational cohort study designed to assess efficacy of STE for assessment of PIVD and PICMP. Sixty subjects with high-grade atrioventricular block and Complete heart block scheduled to undergo right ventricular apical pacing were enrolled for the study.

Eligible patients were aged 18 years or over, had preserved LVEF (>55%) and second-degree or third-degree atrioventricular block. All patients were required to provide written informed consent for inclusion.

Exclusion criteria were pregnancy, myocardial infarction or coronary revascularization within prior 3 months, atrial fibrillation (AF), hemodynamically significant valvular heart disease (moderate in severity), Cardiomyopathy, LVEF < 55%, New York Heart Association (NYHA) functional class III or IV, significant respiratory disease, history of carcinoma within 5 years, autoimmune disorders, rheumatoid arthritis or treatment with disease modifying drugs.

All clinical investigations were conducted according to the principles expressed in the Declaration of Helsinki. Written informed consent was obtained from all participants. Baseline evaluation consisted of: (i) assessment of NYHA functional class (ii) 12-lead electrocardiogram (ECG) (iii) standard transthoracic echocardiography. The protocol required that patients undergo standardized implantation of a permanent pacemaker with positioning of the ventricular lead at the RV apex.

### 2.1 Image acquisition

Standard two-dimensional echocardiography was performed before and after pacemaker implantation (baseline, 1 month and 12 months) using an XARIO 200 PLATINUM ultrasound system with a 1–5 MHz broadband phased-array probe (TOSHIBA). Two dimensional LVEF were measured using Simpson's biplane method. PIVD was defined as an absolute decline in LVEF by 5% points. PICMP was defined as a reduction in LVEF to < 45%. A single experienced Operator who was blinded to the pacing interrogation results performed all echocardiographic analysis.

### 2.2 Speckle-tracking strain analysis

Strain was evaluated off-line from digitally stored images using all advanced software package (cardiac wall motion quantification (CMQ); ToshibaMedical Systems). Longitudinal strain for individual myocardial segments was measured from the apical four-chamber, two-chamber and long axis views (16 segment AHA/ASE model). GLS score was assessed at baseline, 1 month and 12 months. Cut off value for LV dysfunction was GLS score of -14.5.

### 2.3 Statistical analysis

For statistical analysis data were entered into a Microsoft excel spreadsheet and then analyzed by SPSS (version 27.0; SPSS Inc., Chicago, USA) and GraphPad Prism version 5.P-value ≤0.05 was considered for statistically significant.

## 3 Result and analysis

All patients had almost similar baseline characteristics at the time of inclusion in the study. GLS score was assessed at baseline, 1 month and 12 months. We found that 18 patients had a drop in GLS score at 1 month. For the purpose of analysis, the patients enrolled were divided into two groups at 1 month and followed up for the next 11 months. Group A comprised of patients with decline in GLS score at 1 month (Table 1). Group B included patients with preserved GLS score at 1 month (Table 2). These patients were then followed up for the next 11 months and analyzed for various parameters at 12 months (Table 3).

**Table 1:** Data of Patients with Decline in GLS score at 1 month.

No.	Age [Y]	Sex	Baseline LVEF (%)	DM	HTN	IHD	NYHA class I/II	BB	ACEI/ARB	CACB	Diuretics	HR pre pacemaker	High gradeAV block[Mobitz Type II]	CHB	Mean Cum% VP at12 m	LVEF Baseline	LVEF 1 month	LVEF 12 months	Globallongitudinalstrain Baseline	Globallongitudinalstrain 1 month	Globallongitudinalstrain 12 month
1	59	M	55	Y	N	N	Y	Y	N	N	N	37	Y	N	74	55	46	38	−17	−14	−14
2	60	M	60	Y	Y	N	Y	Y	Y	N	Y	39	Y	N	100	56	47	39	−16	−12	−9
3	61	M	58	N	Y	Y	Y	N	Y	Y	N	45	Y	N	75	57	48	40	−17	−13	−10
4	85	M	59	N	Y	Y	N	N	Y	Y	Y	48	Y	N	89	58	49	41	−16	−12	−13
5	82	M	61	N	N	Y	N	N	N	N	N	62	Y	N	100	59	50	42	−16	−12	−12
6	64	M	62	N	N	N	N	N	N	N	N	50	Y	N	100	60	51	43	−17	−14	−11
7	80	M	59	N	Y	Y	Y	N	N	N	N	59	Y	N	100	61	52	44	−17	−12	−10
8	75	M	67	Y	N	N	N	N	N	N	N	39	Y	N	98	62	53	45	−16	−11	−9
9	76	M	60	N	N	N	N	N	N	N	N	66	Y	N	97	63	54	46	−17	−12	−14
10	68	M	53	N	N	N	N	N	N	N	N	38	Y	N	99	64	55	41	−18	−14	−9
11	77	M	55	N	Y	N	Y	N	N	Y	N	65	Y	N	115	65	56	42	−18	−14	−10
12	75	M	65	N	N	N	N	N	N	N	N	64	N	Y	100	66	57	43	−17	−12	−13
13	71	M	60	Y	N	N	N	N	N	N	N	53	N	Y	100	67	58	48	−18	−11	−12
14	80	M	62	Y	N	N	Y	N	N	N	N	52	N	Y	89	55	59	56	−18	−12	−11
15	85	F	55	N	Y	N	Y	N	Y	N	N	41	N	Y	100	56	57	50	−18	−14	−10
16	85	F	55	N	N	N	N	N	N	N	N	40	N	Y	100	57	58	44	−18	−12	−9
17	84	F	64	N	N	N	N	N	N	N	N	39	N	Y	98	66	59	45	−18	−12	−12
18	80	F	67	N	N	Y	N	N	N	N	N	38	N	Y	100	67	46	56	−17	−13	−11

**Table 2:** Data of Patients with Preserved GLS score at 1 month.

No.	Age	Sex	Baseline LVEF (%)	DM	HTN	IHD	NYHA class I/II	BB	ACEI/ARB	CACB	Diuretics	HR pre pacemaker	Second degree AV block	CHB	Mean Cum %VP at 12 m	LVEF 1 month	LVEF 12 months	Global longitudinal strain Baseline	Global longitudinal strain 1 month	Global longitudinal strain 12 month
1	59	M	56	N	N	N	N	N	N	N	N	48	Y	N	10	56	54	−18	−17	−18
2	78	M	64	Y	Y	Y	Y	Y	Y	Y	N	66	N	Y	87	57	63	−17	−16	−16
3	62	M	62	N	Y	N	N	Y	Y	Y	N	50	Y	N	2	58	58	−18	−17	−15
4	66	M	58	N	N	Y	N	N	N	N	N	58	Y	N	3	59	60	−17	−16	−19
5	59	M	61	N	Y	Y	N	N	Y	N	N	57	Y	N	6	60	61	−18	−17	−14
6	60	M	56	N	N	N	N	N	N	N	N	60	Y	N	3	61	62	−17	−16	−15
7	68	M	60	Y	N	N	N	N	N	N	N	49	Y	N	7	62	57	−18	−17	−17
8	69	M	59	N	N	N	N	N	N	N	N	50	Y	N	9	63	54	−17	−16	−18
9	61	M	62	N	N	N	N	N	N	N	N	53	N	Y	55	64	56	−18	−17	−20
10	77	M	60	Y	Y	Y	N	N	Y	Y	Y	57	N	Y	45	65	59	−17	−16	−15
11	78	M	60	Y	Y	N	Y	N	Y	N	Y	54	Y	N	52	61	61	−18	−17	−15
12	84	M	66	Y	N	N	N	N	N	N	N	59	Y	N	1	62	63	−17	−16	−15
13	80	M	66	N	Y	Y	Y	N	Y	N	N	61	N	Y	65	63	62	−18	−17	−18
14	70	M	66	N	Y	N	N	N	N	N	Y	62	N	Y	100	64	60	−17	−16	−15
15	72	M	63	Y	Y	N	N	N	N	Y	N	64	N	Y	85	56	60	−18	−17	−19
16	74	M	59	N	Y	N	N	N	Y	Y	N	65	Y	N	47	57	54	−17	−16	−15
17	66	M	58	Y	N	N	N	N	N	N	N	56	Y	N	32	58	56	−18	−17	−15
18	69	M	62	N	Y	N	N	N	Y	Y	N	59	Y	N	26	61	59	−17	−16	−17
19	71	M	60	Y	N	Y	N	N	N	N	N	48	Y	N	16	62	61	−18	−17	−14
20	73	M	61	N	N	N	N	N	N	N	N	49	Y	N	11	63	62	−17	−16	−15
21	85	M	56	Y	Y	N	Y	N	N	N	N	50	N	Y	80	64	60	−18	−17	−15
22	87	M	59	N	N	N	N	N	N	N	N	53	Y	N	14	56	60	−17	−16	−16
23	65	M	57	N	N	N	N	Y	N	Y	N	57	Y	N	49	60	54	−18	−17	−15
24	64	M	56	Y	N	N	N	N	Y	N	N	57	Y	N	55	61	54	−17	−16	−18
25	70	F	56	N	N	N	N	N	N	N	N	64	N	Y	90	62	63	−18	−17	−14
26	79	F	62	N	N	Y	N	N	N	N	N	57	N	N	01	63	58	−17	−16	−15
27	84	F	61	N	Y	Y	Y	N	N	N	Y	59	Y	N	35	64	60	−18	−17	−17
28	81	F	61	N	N	N	N	N	N	N	N	60	Y	N	26	65	54	−17	−16	−15
29	80	F	62	N	N	N	N	N	N	N	N	49	Y	N	08	61	54	−18	−17	−19
30	70	F	59	N	N	N	N	N	N	N	N	48	Y	N	23	62	63	−17	−16	−15
31	77	F	59	N	N	N	N	N	N	N	N	49	Y	N	34	61	61	−18	−17	−18
32	64	F	58	Y	Y	Y	N	N	Y	Y	N	50	Y	N	17	62	63	−17	−16	−20
33	59	F	59	N	N	N	N	N	N	N	N	52	Y	N	15	61	62	−18	−16	−15
34	59	F	66	N	N	N	N	N	N	N	N	62	Y	N	45	62	60	−17	−17	−12
35	59	F	63	N	N	N	N	N	N	N	N	53	N	Y	76	63	60	−18	−16	−15
36	63	F	65	Y	Y	N	N	N	N	Y	N	60	Y	N	06	64	54	−17	−17	−18
37	87	F	64	N	N	N	N	N	N	N	N	64	Y	N	44	56	56	−18	−16	−15
38	67	F	61	Y	N	N	N	N	Y	N	N	55	N	Y	62	60	59	−17	−17	−15
39	66	F	62	N	Y	N	N	N	N	N	N	52	N	Y	73	56	54	−18	−16	−18
40	60	F	59	N	N	N	N	N	N	N	N	53	Y	N	19	57	56	−17	−16	−15
41	70	F	58	N	Y	N	N	N	N	N	N	49	Y	N	08	58	59	−18	−17	−19
42	60	F	60	N	N	N	N	N	N	N	N	56	Y	N	30	59	59	−17	−16	−15

**Table 3:** Comparison between decline in GLS score at 1 month with preserved GLS score at 1 month with follow up results over the next 11 months.

Parameters	Decline in GLS at 1 M [18 patients]	Preserved GLS at 1 M [42 patients]	Statistical significance
Age [mean in years]	74.83	70.28	No
Sex [Male %]	77.8	57.1	No
Baseline LVEF [Average in %]	59.8	60.5	No
1 month LVEF [Average in %]	53.05	60.6	No
12 months LVEF [Average in %]	44.61	58.69	Yes [P – 0.003]
Baseline GLS score	−17.17	−17.5	No
1 month GLS score	−12.55	−16.4	Yes [P – 0.03]
12 month GLS score	−11.05	−16.17	Yes [P – 0.002]
**Comorbidities**			
DM [%]	27.78	30.95	No
HTN [%]	33.3	40.48	No
IHD [%]	27.78	21.43	No
**Medications**			
Beta blockers [%]	11	7.14	NO
ACEI/ARB [%]	22.22	26.19	No
CACB [%]	16.67	19	No
Diuretics [%]	11.11	9.52	No
**Heart failure**			
NYHA CLASS I/II	38.89	11.9	Yes [P – 0.003]
**Indication for pacing**			
Mobitz Type II [High grade AV block]	11 [61.11%]	31 [73.81%]	No
Complete Heart block	7 [38.89%]	11 [26.19%]	No
Average HR before pacing [BPM]	48	55	No
Cumulative % of Ventricular pacing	96.33	35.05	Yes [P – 0.003]

Our study showed statistically significant correlation between reduced GLS score at 1 month and decline in LVEF at 12 months. This association was statistically significant in patients with higher cumulative percentage of pacing. Statistically significant correlation was also found between decline in ejection fraction and heart failure at presentation. There was no correlation between comorbidities like ischemic heart disease, hypertension and diabetes. There was no correlation between type of cardiac medicine intake.

## 4 Discussion

RV apical pacing has been known to cause pacing induced LV dysfunction. It is often clinically silent and detected late. Conventional 2D Echocardiography often fails to detect subtle changes in LV function at an early stage. Yet, there is an utmost need to detect LV dysfunction early in patients with RV apical pacing because the condition can be reversed or the decline in LVEF could be arrested by medications or upgrading to biventricular or physiological pacing.

We conducted our study at IPD & OPD of Cardiology at Anamoy Super specialty Hospital (Burdwan Medical College and Hospital) for over 12 months. We designed a proforma containing various parameters and collected data accordingly. Data collection from patients was done after explaining the purpose of the study and obtaining informed consent of the legal guardian. Most of the patients coming to hospitals were of low or middle socioeconomic status. Consent form was printed in three languages like English, Bengali and Hindi.

We applied strain imaging and calculated the GLS score as early as at 1 month post pacing to detect any decline in LVEF. Our study revealed that Strain imaging not only picked up early decline in LVEF but the results were consistent with decline in LVEF by 2D method at 12 months. Moreover, there was association between high grade AV block and Complete heart block with higher cumulative percentage of ventricular pacing. Heart failure at presentation was also a potential risk factor for developing decline in LVEF in the long run.

NM Sharath Babu et al. ^16^ applied 3D echo with LV strain imaging to detect LV dysfunction associated with RV pacing. Their findings suggested development of PiCMP with cumulative ventricular pacing percentage of >20%. Our study also revealed pacemaker dependency as a potential risk factor for development of RV pacing induced LV dysfunction.

Shaan Khurshid et al. ^17^ suggested more than 40% pacing burden as a potential risk factor for developing PiCMP, a finding corroborated by us also. They also stressed on Pre-pacing QRS duration [>115 ms] as another potential risk marker.

Moustafa Dawood et al. ^18^ stressed on the need for GLS for early detection of pacing induced LV dysfunction. They calculated 1 week GLS for detection of post pacing LV dysfunction. We calculated pre-implantation GLS (Baseline) and then at 1 month and 12 months on follow-up. Our results also suggested the sensitivity of strain imaging over conventional 2D echo for early detection of pacing induced LV dysfunction.

Samir Rafla et al. ^19^ showed Speckle tracking echocardiography as a sensitive tool for early detection of pacing induced LV dysfunction. These cases if detected early could be upgraded to biventricular pacing.

Victoria Delgado et al. ^20^ discussed in depth analysis of the physiology of LV dysfunction due to RV pacing. By speckle tracking strain imaging he could show the distortion in LV mechanics particularly the loss of LV contraction synchrony and LV longitudinal twist and rotation being primarily affected. We detected similar findings during our study.

## 5 Conclusion

Our study revealed that Strain imaging based GLS score is a sensitive tool to detect RV apical pacing induced LV dysfunction. We could detect drop in GLS score as early as one month when conventional 2D Echocardiography failed to do so. Pacing induced LV dysfunction was more pronounced in pacemaker dependent patients with higher cumulative percentage of ventricular pacing. However, these findings need to be corelated in larger randomized controlled trials.

## Declaration of competing interest

The authors declare that they have no known competing financial interests or personal relationships that could have appeared to influence the work reported in this paper.
